# Some Insights on Grassland Health Assessment Based on Remote Sensing

**DOI:** 10.3390/s150203070

**Published:** 2015-01-29

**Authors:** Dandan Xu, Xulin Guo

**Affiliations:** Department of Geography and Planning, University of Saskatchewan, Saskatoon, SK S7N 5C8, Canada; E-Mail: dax890@mail.usask.ca

**Keywords:** ecosystem, grassland health assessment, grassland monitoring, remote sensing

## Abstract

Grassland ecosystem is one of the largest ecosystems, which naturally occurs on all continents excluding Antarctica and provides both ecological and economic functions. The deterioration of natural grassland has been attracting many grassland researchers to monitor the grassland condition and dynamics for decades. Remote sensing techniques, which are advanced in dealing with the scale constraints of ecological research and provide temporal information, become a powerful approach of grassland ecosystem monitoring. So far, grassland health monitoring studies have mostly focused on different areas, for example, productivity evaluation, classification, vegetation dynamics, livestock carrying capacity, grazing intensity, natural disaster detecting, fire, climate change, coverage assessment and soil erosion. However, the grassland ecosystem is a complex system which is formed by soil, vegetation, wildlife and atmosphere. Thus, it is time to consider the grassland ecosystem as an entity synthetically and establish an integrated grassland health monitoring system to combine different aspects of the complex grassland ecosystem. In this review, current grassland health monitoring methods, including rangeland health assessment, ecosystem health assessment and grassland monitoring by remote sensing from different aspects, are discussed along with the future directions of grassland health assessment.

## 1. Introduction

Grassland, a specific ecosystem, occurs naturally on all continents excluding Antarctica [1]. In grassland, vegetation is dominated by grasses and other herbaceous plants, also including a small amount of shrubs. Grassland covers about one third of the world's terrestrial area [2], with most of the grasslands in semi-arid and arid areas (28% in semi-arid regions, 23% in humid areas, 20% in cold places, 19% in arid districts [1]). Australia, Russia, China, America and Canada are five countries which hold the largest grassland areas [1]. Grassland ecosystem maintains ecological functions, for example, water conservation, carbon storage and site stability (preventing soil exposure and erosion). Moreover, the grassland ecosystem supplies forage for livestock and wild animals without requiring fertilizers input. Grassland ecosystems also provide great landscape views for human beings. Nowadays, grasslands are degrading or have the potential to degrade under the stress of human activities (grazing or recreation), invasive species, or climate changes. Thus, it is necessary to understand grassland health status and dynamics.

The concept of grassland health is crosses ecosystem and health science disciplines [3]. The early definition of ecosystem health is the analogy of human health, for example, treating animal health or plant health as grassland health [4]. However, the definition of grassland health should consider grassland ecosystem as a complex system instead of animal health and plant health, because an ecosystem emphasizes the connection between community processes and the physical environment [5]. It is also not appropriate to just use a keystone species or the productivity of grassland to define grassland health [6]. Keystone species may indirectly present the interaction between them and other species or physical environment in the grassland ecosystem but this does not examine the energy flux, nutrient cycle, productivity, diversity or response capacity to disturbance. Productivity just represents grassland production performance but not biodiversity, the connection between species or between species and their physical environment or resilience of the ecosystem. Costanza and Mageau [7] defined ecosystem health as “a comprehensive, multiscale, dynamic, hierarchical measure of system resilience, organization, and vigor”. Vigor refers to the throughput or productivity of ecosystems [8]. Organization represents not only diversity but also interactions between system components [8,9]. Holling defined resilience as “the ability of a system to maintain its structure and patterns of behavior in the face of disturbance” [10].

Grassland health assessment (GHA) is an approach for evaluating grassland health qualitatively or quantitatively. GHA is not only a comprehensive grassland monitoring approach, but also a bridge of grassland research and grassland management. To make appropriate grassland management policy, the priority is to evaluate grassland health condition because grassland management is different for healthy grassland, sub-healthy grassland and unhealthy grassland. Suitable tourism and grazing strategies could be executed in healthy grassland which maintains its organization and resilience [11] and provides a quantity of functions without requiring fertilizer input, such as high productivity, site stability, capture and beneficial release of water [12], grazing and recreation; while sub-healthy grassland needs to be protected and conserved, and efficient recovery management should be implemented in unhealthy grassland. The objective of this review is to survey and summarize current methodology, challenges and future directions for GHA.

## 2. Current Methodology and Challenges for Grassland Health Assessment

In the literature, rangeland health assessment (RHA) and ecosystem health assessment (EHA) are the two applicable methods for GHA, and the approach of remote sensing started to be used to evaluate ecosystem health until the EHA came to a stage of quantitative assessment. Previous studies of grassland monitoring using remote sensing provide evidence that remote sensing, with multi-temporal and multi-spatial images, has the potential to evaluate the indicators of grassland health in multiple spatial and temporal scales. Therefore, research related to GHA includes three major fields: (1) rangeland health assessment; (2) ecosystem health assessment; and (3) grassland monitoring using remote sensing. The first major field emphasizes the functionality of grasslands (e.g., grassland for grazing); the second major field includes different methods which are suitable for various ecosystems including grassland, forest, cropland and marine ecosystem; and the third major field focuses on grassland health evaluation in different biophysical aspects.

### 2.1. Rangeland Health Assessment

The concept, framework and methodology of RHA provide tools for the evaluation of grassland health condition. The methodology of RHA mainly has three directions. The first one is using key species, including wildlife [13,14], vegetation and soil crust [15], as the indicators of RHA, which only provides the general sense of rangeland health condition. The second one is selecting the vegetation level or soil level to monitor rangeland health [12,16,17]. For this concept of RHA, the wildlife, climate condition and the interaction between vegetation level, soil level, animal level and climate level were not under consideration. The third one is a comprehensive modeling based on rangeland structure and functions [18–20], because rangeland health research has paid more attention to how efficiently rangeland could maintain the structure and functions for grazing [21]. In research applying the third direction of RHA, three main methods—interpreting indicators of rangeland health (IIRH), landscape function analysis (LFA), and RHA—were used by University of Alberta to monitor rangeland health condition [21–23], Table 1. All three RHA systems are mainly functions and structure-orientated assessment systems. However, most of the grassland ecosystems are human dominated ecosystems, and human activities have altered the ecosystem structure [9]. In addition, the three methods are all rankings based, instead of quantitative assessment based. Ranking is affected by different people's observations, making it difficult to achieve a consistent assessment spatially or temporally. The difficulty in evaluating the spatial variation of rangeland health at a higher spatial scale (e.g., pixel scale) is another disadvantage of RHA.

### 2.2. Ecosystem Health Assessment

Early research of EHA includes using keystone species (lichen or some animals) as indicators of ecosystem health [14,31,32], emphasizing specific aspects of ecosystem health (e.g., diversity–abundance relationship [33], resilience [34] or energy flow [35]), and ranking current ecosystem health by comparing current ecological status with the reference status [36,37]. EHA has been developed as a quantitative assessment and has been widely used since Costanza *et al.* [38] developed an overall ecosystem health index, HI = V × O × R., where “Vigor (V) means ecosystem primary production; organization (O) means species diversity and numbers of interactions between system components; resilience (R) means system capacity to maintain structure and function in the presence of stress” [9,39]. Remote sensing technologies bring EHA based on Costanza's concept into a new stage of quantitative assessment [40,41]. However, three ecosystem health indicators (vigor, organization and resilience) were weakly estimated in the literature (Table 2), even with the help of remote sensing which benefits large-scale monitoring and temporal change detection in EHA.

Normalized Difference Vegetation Index (NDVI) was commonly used for evaluating “Vigor” which can be evaluated by ecosystem productivity. NDVI has been used to measure net primary productivity by empirical or physical models [42–44]. However, NDVI represents the amount of green vegetation, including forage, weed, shrub and so on, so high NDVI does not exactly mean high grassland primary productivity because of the influence of weeds and noxious grass. “Organization” was usually measured by diversity (biodiversity or landscape diversity) or vegetation coverage. Previous studies evaluated diversity to present “Organization”, but failed to address the interactions between ecosystem components.

The assessment of “Resilience” varied from different studies, including variation of different factors, pressure of ecosystem (e.g., grazing), or limitation factors (e.g., slope). Commonly, pressure (e.g., livestock capacity, number of livestock) was used to evaluate “Resilience”. Evidence shows that pressure changes ecosystem resilience. Whitford *et al.* [34] tested the resistance and resilience of stressed and relatively unstressed ecosystems to drought by the survivorship of the perennial species in the Chihuahuan desert grassland, and the results show that the less stressed ecosystem has higher resistance to drought than the heavily stressed ecosystem. Pervious pressure influences current ecosystem status, and current pressure may affect the future ecosystem state. However, resilience could not just be estimated by current pressure; instead, resilience means an ecosystem's ability to remain in its current state and return to this state when ecosystem is under stress [49].

### 2.3. Grassland Monitoring Using Remote Sensing

Remote sensing is defined “as the art and science of obtaining information about an object without being in direct physical contact with the object and is a scientific technology that can be used to measure and monitor important biophysical characteristics and human activities on Earth” by Jensen [50]. After the first Landsat satellite was launched in 1972, satellite images were available for ecological researchers [51]. The application of remote sensing techniques in grassland monitoring has a history from the 1980s [52,53]. A number of studies integrated grassland monitoring (including monitoring vegetation, animal, soil and environment) and remote sensing together (Table 3). Among all the studies (1057 studies from 1984 to 2015, searched from Web of Science), 70% studies in the field of grassland monitoring using remote sensing focused on vegetation level, 29% were in the animal level, 30% were in soil level and 25% were in environment level (Table 3). Among five countries which hold the largest areas of grasslands, 14.1% studies in the field of grassland monitoring with remote sensing were for grasslands in Australia, 4.3% for grasslands in Russia, 29.3% for grasslands in China, 38% for grasslands in America and 14.3% for grasslands in Canada. These large scale grassland monitoring researches illustrate different aspects of grassland monitoring by remote sensing technology, which assist in measuring the indicators of EHA (vigor, organization and resilience). However, they lack a comprehensive and consistent approach for monitoring grassland ecosystems [54]. In addition, among all the grassland monitoring research by remote sensing approaches, most have focused on unhealthy grassland, for instance, grassland degradation (desertification and salinization). In fact, evaluation of healthy grassland is as important as that of unhealthy grasslands because healthy grassland can still maintain its functions to human beings, including livestock forage, protecting and conserving soil and water resources, and furnishing a habitat for wild animals and supplying unique landscape views [55].

Scale is often an issue for the application of remote sensing on grassland monitoring. Human activities and physical processes cause the variations in grassland ecosystems in specific temporal and spatial scales [105]. Therefore, various scales in grasslands have a large impact on grassland monitoring modeling including GHA. Different grassland biophysical parameters require different scales to capture their spatial or temporal variation [106,107], which is because the determinant factors of biophysical parameters are on different scales and even the interactions of different determinant factors create new scales. It requires the methodology of GHA including analyses on multiple spatial and temporal scales to address the scale issue. Remote sensing is a multiscale approach for modeling and analysis [108], which overcomes the scale issue for ecological studies. However, grassland variations in specific temporal and spatial scales caused by human activities, physical process and interactions among different determinant factors may not be explained by the temporal and spatial resolutions of current remote sensing systems [105]. Hence, it is still a challenge to find suitable scale (both temporal and spatial scales) thresholds for GHA modeling at the level of indicators, the level of the interaction among indicators and the level of the entire comprehensive model.

### 2.4. Overall Challenges of Current Methodology

**Figure 1.**
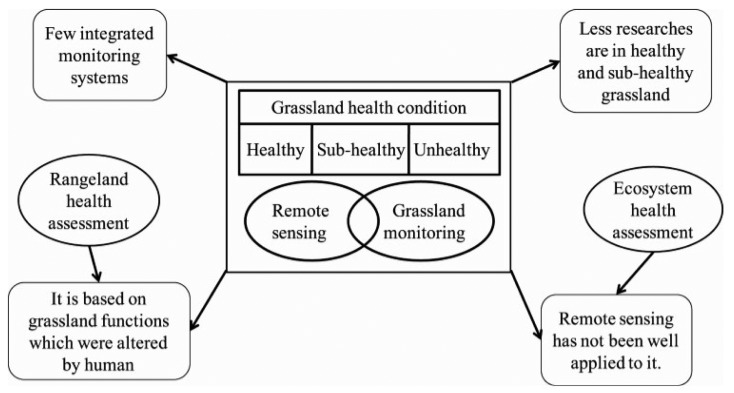
Challenges of current methodology for grassland health assessment.

The overall challenges of GHA concern four different aspects (Figure 1): (1) there has been a wide use of RS technology in grassland monitoring, however, few studies integrated different aspects of grassland monitoring and established a comprehensive and consistent grassland monitoring system; (2) among those grassland monitoring studies, most were about unhealthy grassland, such as grassland desertification and salinization. Very few studies focused on healthy grassland or sub-healthy grassland. Evaluation of these three categories of grassland health condition is necessary because grassland management varies from different grassland health conditions; (3) previous grassland health studies were mostly in RHA, which was evaluated by grassland functions and structures. However, human activities altered grassland functions and structure greatly in both tamed grassland and natural grassland. So, it is requisite to establish a GHA system based on grassland characteristics instead of grassland functions and structure; (4) EHA based on ecosystem attributes could be well applied to grassland health evaluation, which could overcome the gap of RHA. However, remote sensing application has not been well applied in this field.

## 3. Future Directions of Grassland Health Assessment by Remote Sensing

### 3.1. Application of Ecosystem Health Assessment Concepts on Grassland Health Assessment

**Figure 2.**
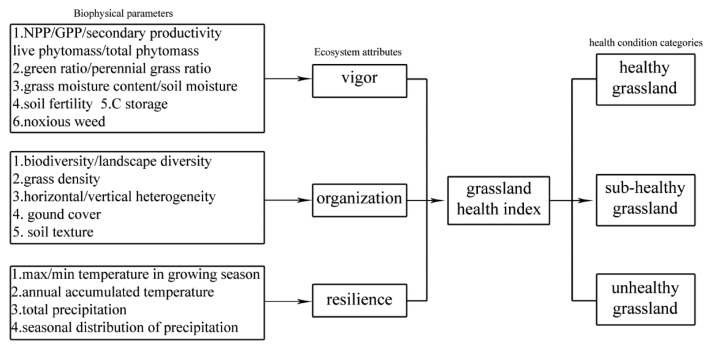
The framework of future directions of grassland health assessment.

The future direction of GHA could be a comprehensive model which links grassland monitoring by remote sensing approaches and the concept of EHA based on three ecosystem attributes (vigor, organization and resilience). Ecosystem health indicators could be measured by biophysical parameters (for example, grassland productivity represents vigor of grassland ecosystem), and those biophysical parameters will be estimated by remote sensing of which there are various methods in the field of grassland monitoring (Figure 2). This will help to overcome the lack of a comprehensive examination of grassland ecosystems in previous studies whereby most research has focused on different aspects of grassland monitoring studies. This direction of GHA will generate a consistent grassland health monitoring system based on natural attributes of grassland ecosystems as well. Based on remotely sensed datasets with multi-temporal and multi-spatial scales, grassland health condition would be comparable quantitatively, even on a pixel scale. More importantly, the dynamics of grassland health conditions would be examined by temporal analysis. The changes of grassland health conditions will provide foundations for grassland managers because health condition dynamics indicate the impacts of grassland management.

### 3.2. Estimation of Ecosystem Health Attributes by Biophysical Parameters

Description of these ecosystem attributes is easier than measurement [8]. In the previous studies, for example, vigor was measured by a normalized difference vegetation index [46] which was extracted directly from remote sensing images. In this case, there would be a large gap between information obtained from images and from ecosystem attributes. Thus, measurement of these three ecosystem attributes could include various biophysical parameters related to the definition of vigor, organization and resilience representatively (Figure 2, Table 4).

Vigor could be measured in term of metabolism, productivity (net/gross primary productivity and second productivity), soil fertility and yield [41]. According to the definition of vigor, it is measured by primary productivity indirectly. Primary productivity contains the productivity of all the green vegetation, including forb, grass, annual weed, shrub, noxious vegetation and so on. Ecosystem vigor excludes the productivity of noxious vegetation because noxious vegetation has no contribution to ecosystem energy flux. However, noxious vegetation still has ecological value for reducing soil erosion (maintaining site stability). If two or more biophysical parameters have high correlation, they cannot be used to evaluate an ecosystem attribute together. Some of the correlations could be detected from the definition of biophysical parameters, for example, gross primary productivity, net primary productivity and biomass; some of the correlations can be tested by statistical methods (correlation analyze); other correlations can be measured by the estimation method of the biophysical parameters, for instance, LAI and biomass, which are both estimated based on vegetation index. Then, one parameter can be selected among all the variables which have high correlation based on the literature and the availability of data.

Measurement of organization includes both diversity and interactions between system components based on the definition of organization [8,9]. Organization was evaluated by landscape diversity or vegetation cover in previous studies [46,47]. Actually, organization includes diversity in different spatial scales (biodiversity, plant form diversity or landscape diversity) and also the connection between ecosystem components. Unlike plant form diversity or landscape diversity [116], biodiversity is really hard to measure by remote sensing approaches [117]. Long term species competition forms current status of diversity and species competition is the ecological connection at the species level. The connections among the whole ecosystem can be represented by energy flux and nutrient cycling. Remote sensing has high potential to evaluate both energy flux [118] and nutrient cycling [119–121] proved by previous studies. Correlation tests need to be applied for the biophysical parameters (e.g., heterogeneity and landscape diversity) as well in organization.

The biophysical parameters in vigor and organization can be simulated by field data and remotely sensed images. Field data have limitations that it does not cover the total study area. Therefore, equations that link the field data and remotely sensed data are formed based on the available field data and its matching data extracted from remote sensing images. In the literature, the quantity of simulation models were developed for biophysical parameter extraction, including NPP simulation model, ground cover evaluation based on soil line, C3 and C4 percentage cover by vegetation line, structure extraction by texture analysis, *etc.* When choosing remotely sensed images, the spatial and temporal variations of required biophysical parameters need to be taken into consideration. Also, the scale of field design needs to be correlated with the spatial resolution of remotely sensed data. In addition, spectral resolution should also be considered when selecting remote sensing data sources because a certain biophysical parameter has a specific requirement for the spectral signature; for example, the unique spectral signature for green grass is the red edge. The balance between spatial, temporal, radiometric and spectral resolution is also very important because one remote sensing data source might not meet all the requirement of all these resolutions.

Resilience means ecosystem ability to remain its current state and return to this state when ecosystem is under stress [49], which can be assessed as a system's counteractive capacity [9]. It cannot be measured directly. Because ecosystem is different from a spring, we cannot add different levels of stresses to an ecosystem to measure the biggest stress strength an ecosystem could endure. It can be calculated indirectly by environmental duress, which refers to restriction factors for plant growth in the environment [39]. The environmental data will be collected from meteorological observatory. If the value of biophysical parameters in resilience part (Table 4) is less than normal requirement of grass growth, those biophysical parameters are restriction factors.

### 3.3. Grassland Health Assessment Modeling

Besides the measurement of the biophysical parameters in three ecosystem attributes (vigor, organization and resilience), the comprehensive GHA modeling also includes the sensitivity test of the indicators, and the weights assigned to the biophysical parameters and ecosystem attributes. Weight refers to the relative importance of each factor or each index [122], which will be used to calculate grassland health index in this case. In the literature, two main kinds of methods were used to assign weight for factors, parameters or indices. One is mathematic method totally based on the data characteristic and has no requirement of human experience or pre-knowledge. Weight determination methods in this category are the factor analysis weight method, principle component weight method, entropy weight method and information weight method. The factor analysis weight method and principle component weight method are similar, which consider the accumulative contribution of all the parameters in a certain dataset, and parameters with higher accumulative contribution would be assigned higher weight. Entropy weight method according to the randomness of a system assigns higher weight to a parameter with lower entropy value. The information weight method assumes that parameters with higher coefficient of variation have high amount of information, so these parameters have high weight. Another category of weight determination method is expert consultation weight method, including the Delphi method and analytic hierarchy process (AHP). This kind of weight determination method requires expert consultation information, it means experts in a certain field (in this study, grassland experts) assign initial weight for each parameter. The Delphi method deals with the initial weights with simple mathematic method, for example, average value or mode value of initial weights for each parameter, while AHP which combines both quantitative and qualitative criteria would check the consistency of initial weights of all the experts [123]. Sensitivity test is necessary for measuring how the value of grassland health index changes by changing the indicator values at both the ecosystem attributes' level and biophysical parameters' level.

### 3.4. Three Grassland Health Conditions (Healthy, Sub-Healthy and Unhealthy)

It has the potential to assess grassland health condition not only in a quantitative way but also in a qualitative direction. It is important to divide grassland health condition into three categories (healthy, sub-healthy and unhealthy in Figure 2) because grassland management varies from these three grassland health conditions. While healthy grasslands provide ecological and economical services sustainably, sub-healthy grasslands need to be conserved and unhealthy grasslands need to be restored. Cluster analysis is a classification method that stratifies a set of objects into two or more groups according to the similarity of the objects [124], which has the potential to classify grassland health conditions. Cluster analysis is powerful not only in separating groups which have nature breaks but also in producing clusters when the dataset has no cluster structure [125]. Cluster analysis has been widely used as a classification method to separate plant species in plant science [126], as a statistic method to analyze remotely sensed imagery [127], and as a common method to identify environment patterns and ecoregions in earth science [124,128].

## 4. Conclusions

In conclusions, RHA and EHA are both comprehensive monitoring systems. Grassland health research has mostly used RHA to evaluate grassland functions and structure, but the structure and functions are altered by human activities. EHA based on ecosystem attributes (vigor, organization and resilience) could be well applied in GHA. EHA is not widely applied on a global scale because it has not yet incorporated much remote sensing; however, the concept based on ecosystem attributes could be well applied in GHA. Therefore, the combinations of EHA concepts, grassland monitoring of different biophysical parameters and remote sensing approaches will comprise the future direction of GHA in both quantitative and qualitative ways. More importantly, quantifying and qualifying grassland health conditions on multi-spatial and multi-temporal scales benefits grassland management and economy.

## Figures and Tables

**Table 1.** Strength and limitations of three Rangeland Health Assessment (RHA) methods.

**RHA Methods**	**Indicators**	**Strengths**	**Limitations**
IIRH [24–27]	soil/site stability	It is a comprehensive RHA system considering plant community, soil property and biotic environment using 17 secondary indicators.	It is rangeland function and structure orientated It is a ranking assessment instead of quantitative assessment.
	hydrological function		
	integrity of biotic community		
LFA [28–30]	landscape organization (e.g., patches and inter-patches)	It is an innovation direction for rangeland functioning (e.g., runoff) assessment based on grass patches and inter-patches.	It mainly focuses on landscape functioning, lacking of estimation for plant community, ecosystem connections and resilience. It is a ranking assessment instead of quantitative assessment.
	soil surface (e.g., perennial vegetation cover, litter, crust brokenness, soil erosion and surface roughness)		
RHA by University of Alberta [21]	integrity and ecological status	It integrates ecological status, rangeland structure and functions for health assessment	It is a ranking assessment instead of quantitative assessment.
	community structure		
	hydrological function and nutrient cycling		
	site stability		
	Noxious species		

**Table 2.** Previous research of ecosystem health assessment using remote sensing.

**Previous Studies**	**Ecosystem Health Indicators**	**Secondary Indicators**
Chen and Wang [45]	Vigor	Annual max NDVI
	Pressure	Actual number of livestock
	Resilience	The ratio of NDVI max to min
Suo *et al.* [46]	Vigor	NDVI
		Erosion modulus
		Depth of runoff
	Organization	Landscape diversity
		Uniform of erosion
		Uniform of runoff
	Resilience	Landscape richness
		Variation of erosion
		Variation of runoff
Chen *et al.* [47]	Vigor	Annual average NDVI
	Organization	Vegetation cover
		Vegetation centriod movement
	Resilience	Slope
Li *et al.* [48]	Vigor	Aboveground biomass
		Photosynthetic rate
		Organic matter
		Bulk density
	Organization	Biodiversity
		Primary species proportion
	Resilience	Vegetation cover
		Grazing capacity

**Table 3.** Grassland monitoring by remote sensing in four different levels.

Vegetation level	Grassland productivity evaluation [53,56–60]
	Grassland degradation [61–64]
	Grassland classification [65,66]
	Grassland reclamations [67,68]
	Vegetation dynamics [69–72]
	Canopy or vegetation cover [73,74]
	Grassland carbon flux and storage [75–78]
	Species invasion in grassland [79–81]
	C3 and C4 grasses distribution [82–85]
	Grassland management impacts [86]
	Grassland response to disturbance or stress (human activities [87], grazing [88], fire [89], climate change [90,91])
Animal level	Livestock carrying capacity [92,93]
	Grazing intensity monitoring [88,94]
	Habitat mapping [95]
	Population decline of wildlife [96]
Soil level	Soil erosion and soil conservation [97]
	Soil organic carbon [98]
	Soil moisture [99,100]
	Soil crust [101]
Environment level	Evapotranspiration monitoring [102,103]
	Groundwater level estimation [104]

**Table 4.** Biophysical parameters for estimating ecosystem attributes.

**Ecosystem Attributes**	**Biophysical Parameters**	**Explanation**
vigor	gross primary productivity (GPP)	the total energy fixed by plant through photosynthesis in a given length of time [109]
	net primary productivity (NPP)	NPP = GPP − respiration (by plant)
	secondary productivity	total biomass generation by heterotrophs [110]
	live phytomass	dry matter of living plants including aboveground biomass and belowground biomass [111]
	total phytomass	total plant biomass
	green ratio (GR)	the relative amount of live and dead materials
	perennial grass ratio	the percentage of green perennial grasses in total green grasses
	grass moisture content	(weight of living grass − weight of dried living grass)/weight of living grass
	soil moisture	(weight of soil − weight of dried soil)/weight of soil
	soil fertility	rich of nutrients for basic plant nutrition, including N, P and K [112]
	carbon storage	the storage of carbon in grasses and soil [113]
	noxious weed	the percentage of noxious weed of total grasses
organization	biodiversity	the degree of variation of grasses [114]
	landscape diversity	the degree of variation of landscape patterns
	grass density	percentage of grasses per unit volume
	horizontal heterogeneity	grass diversity in the horizontal direction
	vertical heterogeneity	grass diversity in the vertical direction
	ground cover	the percentage cover of grass, shrub, forb, bare soil, rock and ground soil crust (moss and lichen)
	soil texture	the relative proportion of different grain sizes of soil mineral particles [115]
	nutrient cycling	Normally, plants take the nutrient from atmosphere and soil. When plants are consumed by grazers, nutrient is transfer from plants to animals. After animals died, the nutrient is decomposed by decomposed system and become inorganic matter which can be taken by plants again
	energy flow	Energy flows from solar energy to net primary productivity, then to secondary productivity, finally transferred to decomposer system
resilience	maximum temperature in growing season	the max temperature in grass growing period
	minimum temperature in growing season	the min temperature in grass growing period
	annual accumulated temperature	the accumulated temperature in one year
	total precipitation	total precipitation in one year
	seasonal distribution of precipitation	the percentage of precipitation in grass growing season or brown season
